# Effects of pelleted timothy hay on pair-housed Holstein calf performance

**DOI:** 10.1093/jas/skag044

**Published:** 2026-02-16

**Authors:** Gillian D Plaugher, Melissa C Cantor

**Affiliations:** Department of Animal Science, Penn State University, University Park, PA 16802, United States; Department of Animal Science, Penn State University, University Park, PA 16802, United States

**Keywords:** body weight, dairy calf, hay, growth, social housing

## Abstract

This randomized control trial evaluated the effects of feeding pelleted hay to paired dairy calves on body weight (BW), average daily gain (ADG), calf starter dry matter intake (DMI), and solid feed efficiency (FE) up to one week after weaning (75 d of age; mean ± SD). Holstein heifer calves (*n* = 32 pairs; 64 calves) were enrolled at pairing (5 ± 3 d of age; referred to hereafter as day 0 of the study) and randomly assigned in blocks of four pairs to a control (CON; no hay) or treatment (HAY; timothy hay). All calves received 7.4 L/d of milk replacer (MR; 22% crude protein [CP], 20% fat) until 56 d, 3.2 L/d from day 57 to 62, and were weaned on day 63. The HAY group had a trough with pellets until day 59, then they were transitioned to long-stem timothy hay until study completion on day 70 (7 d postweaning). Starter and hay intake and refusals were recorded daily. We measured BW twice weekly until 30 d, then weekly, and a final BW was recorded on day 70. We calculated the average pair starter DMI, solid FE, ADG, and BW by period where period 1 = birth to day 26, period 2 = day 27 to 60, period 3 = day 61 to 67, and period 4 = day 68 to day 75 ± 3 d of age. We used mixed linear regression models to assess the impact of HAY, period, and the HAY × period interaction on ADG, BW, solid FE, and starter DMI, adjusting for birthweight, season, serum total protein, and period with period as a repeated measure, calf as the subject and pair nested within block as a random effect. We used Tukey adjustments to correct for multiple mean comparisons. There was no association of HAY with ADG (CON; 0.88 ± 0.03 vs. HAY; 0.88 ± 0.03 kg/d), starter DMI (CON 2.68 ± 0.15 vs. HAY 2.50 ± 0.14 kg/d), or solid FE (CON 0.65 ± 0.07 vs. HAY 0.73 ± 0.07). We suggest that pelleted timothy hay offered to paired calves warrants further research on behavior and health outcomes, and it does not impact calf starter intake, BW, or average daily gains.

## Teaser

Pair-housed Holstein heifer calves fed timothy hay during the preweaning period had similar growth performance, solid feed dry matter intakes, and feed efficiency to control calves.

## Introduction

Calves that are fed at least 1 kg/d on a dry matter (DM) basis of milk replacer (MR) have improved growth performance (Rosenberger et al. 2017), but the success of this diet depends on sufficient starter intakes of at least 1.36 kg/d (Drackley 2008) and adequate rumen development before weaning (Roth et al. 2009). This is why many producers restrict dietary fiber to a small percentage of dry matter intake (DMI) <5% in the calf diet preweaning (NASEM 2021). Restricting fiber facilitates the consumption of calf starter to feed ruminal microbes, leading to butyrate production for early papillae development (Khan et al. 2016). However, the challenge of not offering calves forage is that it predisposes them to acidosis: greater starter intakes during fiber restriction were associated with prolonged periods of a ruminal pH below 4.0 (Yohe et al. 2022). Moreover, a review by Khan et al. (2016) and a meta-analysis by Imani et al. (2017) found that providing at least 10% DM of forage in the diet improved calf average daily gains (ADG). However, all the work reviewed by Khan et al. (2016) and the meta-analysis from Imani et al. (2017) involved studies that fed forage to individually housed calves.

Furthermore, as more producers adopt social housing, reducing the prevalence of cross-sucking is important because it may have a negative outcome on calf welfare (Doyle et al. 2024). Long-stem hay reduces abnormal oral behaviors and increases feeding time in calves (Horvath and Miller-Cushon, 2019; Zhang et al. 2021). This may reduce cross-sucking by prolonging eating and chewing activities and enhancing ruminal fill and buffering. This distinction is important because calves are naturally food neophobic (Costa et al. 2014), and socially housed calves eat more hay than individually housed calves (Hepola et al. 2006). Calves paired in early life benefit from social facilitation, or the presence of another peer, which encourages consumption of solid feed earlier than individually housed calves and may also positively impact growth performance (as reviewed by Costa et al. 2016a). Social facilitation also influences how calves learn to graze and how they select new feeds as safe to eat (Costa et al. 2016b). No researchers have investigated if offering hay affects performance in paired dairy calves, which is important because calf starter DMI needs to be maintained when forage is offered to ensure weaning at a young age (as reviewed by Plaugher and Cantor 2025). It is also important to distinguish work that offers feed to paired calves rather than complex social housing because larger social groups usually have additional enrichments that could discourage abnormal behavior, such as brushes and forage, which facilitates the redirection of abnormal behavior in paired calves (Zhang et al. 2021). Pair-housed calves benefit from social facilitation and likely also have different consumption patterns of forage than individually housed calves. Therefore, we suggest research is needed to elucidate if small socially housed groups of calves (i.e., pair housing) benefit from supplemental forage.

One challenge with testing forage provision in pair-housed dairy calves is deciding which variety and physical form to offer them, as most researchers fed calves individually. In fact, only one study has investigated timothy hay on socially housed calf performance, while it discouraged abnormal oral behaviors, growth performance was increased in the individually housed calves (Horvath and Miller-Cushon 2017). Coverdale et al. (2004) observed that the inclusion of chopped bromegrass hay in the calf diet improved calf body weight (BW), ADG, and FE during the postweaning period. Castells et al. (2012) found that preweaned calves offered roughages other than alfalfa had improved starter DMI and ADG, while these outcomes were not observed in calves fed alfalfa. Grass hays may provide better rumen development advantages for calves compared to alfalfa. For example, Khan et al. (2011) summarized that calves offered chopped orchard grass hay and fed higher planes of milk (=1.0 kg/d DM or 8 L/d of waste milk) had greater reticulorumen weights and ruminal pH than calves not offered hay, with no difference in papillae width, length, or number of papillae per square cm (Khan et al. 2011). Alfalfa is higher in % crude protein (CP) than timothy, oat, or orchard grass hay (NASEM 2021), which can be a concern for limiting feed efficiency (FE) in calves. In fact, alfalfa was associated with decreased FE during the preweaning period when compared to other forage varieties in calves (Imani et al. 2017). These findings warrant further investigation of grass hay and its effects on calf ADG, BW, and FE.

Chopped hay or long-stem hay have variable nutrient quality depending on what it is harvested, such as the total CP content, neutral detergent fiber (NDF), and acid detergent fiber (ADF) (NASEM 2021). If calves are offered a late cut forage, such as straw, the fiber content may increase gut fill and discourage calf starter intakes (Hill et al. 2008). Pellets also have a more consistent nutrient composition for calves (Molaei et al. 2021). Alfalfa hay is the only pelleted hay that has been studied in preweaned calves, but benefits are dependent on how the pellets are delivered to calves (Kincaid 1980; Jahani-Moghadam et al. 2015; Molaei et al. 2021). For example, Kincaid (1980) observed growth benefits when feeding pelleted alfalfa at a 20% inclusion rate in the concentrate mixture to calves, but not when offered separately *ad libitum*. Jahani-Moghadam et al. (2015) observed the opposite, there was no association of feeding pelleted alfalfa with calf performance when offered as a total mixed ration component of the diet. Molaei et al. (2021) also fed pelleted alfalfa as a total mixed ration to calves; there was no growth benefit, and the pellet-fed calves had compromised neutral fiber component digestibility compared to calves offered chopped hay. These results suggest that other forages should be investigated as a potential source of pelleted forage for calves.

While the effects of alfalfa hay on calf performance have been investigated, there is no research on the effects of pelleted feeding timothy hay on calf performance. Research on pelleted alfalfa has focused on comparing individually housed calves offered the forage against those without forage access (Jahani-Moghadam et al. 2015; Molaei et al. 2021). No study has evaluated pelleted hay feeding in socially housed calves. However, it is known that pair-housing can improve growth performance and solid feed intakes(Plaugher and Cantor 2025). Therefore, the objective of this study was to assess the effect of timothy hay in pelleted and long-stem forms on pair ADG, BW, starter grain DMI, solid FE in pair-housed Holstein calves. We hypothesized that feeding timothy hay would increase paired calf BW, ADG, and solid FE, while having no impact on calf starter DMI.

## Materials and methods

All animal procedures were approved by the Penn State University Institutional Animal Care and Use Committee (IACUC: PROTO202302536) prior to study commencement.

### Study population and power analysis

A power analysis was calculated to ensure that enough pairs were followed to capture a targeted difference in growth between HAY vs. CON pairs. According to Bahmanpour et al. (2023), calves were expected to have a preweaning ADG of 0.58 kg/d, and calves fed forage achieved an ADG of 0.67 kg/d. At an expected standard deviation of 0.08 kg/d (our average barn variability in growth) at 80% power and with 95% confidence, we required 12 pairs per treatment to detect a statistical difference. We chose to use the variance of ADG from our facility to power this study to ensure that the growth variation within a pair was part of our power calculation. This was done because research offering forage to calves in pair housing was not conducted at the time of the power analysis. We enrolled 16 pairs per treatment to appropriately evaluate outcomes for a concurrent study.

### Enrollment and management procedures

Every Holstein heifer calf born at the Penn State University Research Dairy that met our enrollment criteria was enrolled in this study from January 21, 2024, until the final pair of calves completed the study on November 5, 2024. Our enrollment criteria were that calves weighed ≥33 kg at birth and that calves did not have diarrhea at a fecal consistency of watery before pairing (Renaud et al. 2020). Calves were raised in an indoor barn in custom adjacent individual plastic pens that had solid sides (Calf-Tel, WI, USA) after birth until they drank their milk bottle unassisted. These pens were custom built to allow space allowance per calf at 3.71 m^2^. The backside of the pen was an open wire mesh for airflow and was positioned such that no other calves could contact one another. Calves were paired (*n* = 32 pairs) to an adjacent calf at 5 d (± 3 d) of age (mean ± SD) by pulling the adjacent siding. Fans were hung above the calf pens every 5 meters and turned on when the temperature humidity index (THI) was above 70° using an indoor temperature monitor (Kestrel Instruments, PA, USA). All calves completed the study at 7 d post-weaning at 75 (± 3 d) of age. All calves that met enrollment criteria were paired and assigned to either the control (CON) or treatment (timothy hay; HAY) diet in eight randomized blocks of four to balance treatments over the duration of the study. Randomization was performed in Microsoft Excel (version 16.78, Microsoft Corp., USA). All pairs were randomly assigned to blocks and treatments before the commencement of the study by the first author. Calves were housed in plastic-sided housing in a calf barn with wood shavings for bedding, which was topped off daily.

All calves received 4 L of colostrum (≥22% BRIX) fed by farm staff no later than 6 h after calving. Between 24 to 48 h after receiving colostrum, whole blood samples were collected by one researcher from all calves using manual restraint in their home pen. Jugular venipuncture was completed using a sterile needle and sampling blood into a 10 mL vacutainer tube without anticoagulant (BD Vacutainer, Becton, Dickinson Co. Franklin Lakes, NJ, USA). The blood was allowed to clot at room temperature, and 5 mL of serum was obtained via centrifugation at 3,300 × *g* for 20 min at 25 °C within 1 h of collection. The serum was tested twice for serum total protein using a digital refractometer (Palm Abbe digital refractometer, Misco TM, Solon, OH). The refractometer was calibrated before testing each time with nanopure water.

All calves received an oral paste per manufacturer’s instructions (First Defense, ImmuCell Corporation, Portland, ME, USA) immediately after receiving colostrum to provide antibodies for E. coli F99, Coronavirus, and Rotavirus. All calves were disbudded by trained farm personnel based on availability before step-down weaning ranging from 35 to 49 d of age by hot iron. All calves received a lidocaine corneal nerve block and oral meloxicam, which were administered by BW 15 min before disbudding.

### Feeding management

All calves were fed 1.0 kg/d of milk solids reconstituted to 7.4 L/d (135 g/L) of Land O’ Lakes Amplifier Max (Land O’ Lakes Inc., Arden Hills, MN, USA) MR containing 22% CP and 20% crude fat (4.7 Mcal/kg) by farm staff divided into two feedings (6 a.m. and 3 p.m.) by bottle. The feedings were allocated to each calf’s bottle with a milk shuttle for precise feeding. After pairing, two bottles (Coburn Co., Whitewater, WI, USA) were placed in the bottle holder between milk feedings. Each bottle contained a calf starter with 20% CP and 4% crude fiber (East Gate Feed and Grain, Reedsville, PA). The Braden bottles were provided after pairing, as others have observed that this reduces the duration of cross-sucking bouts among pairs and is seen as a source of enrichment that discourages unwanted oral behavior in calves (Salter et al. 2021). The same calf starter was also provided from birth by two buckets in front of each pen. Calves received 7.4 L/d of MR until 60 d of age, then were step-down weaned by reducing MR to one feeding at 3.5 L/d for 7 d. Calves were completely weaned on day 68 (± 3) d of age and completed the study at 75 d (± 3 d).

### Experimental periods

Since diarrhea affects ADG (Schinwald et al. 2022), and weaning affects growth patterns (Rosenberger et al. 2017), we opted to divide our growth performance into biologically relevant periods where period 1 was the diarrhea risk period from birth to day 26 of age, period 2 was the full milk period from day 27 to 60, period 3 was weaning from day 61 to 67, and period 4 was the 7 d after weaning from day 68 to 75 (± 3 d). We also chose to divide our periods 3, and 4 based on the removal of milk rather than the transition to long-stem hay because of foundational work that found that weaning age and the removal of milk change the growth trajectory of calves independent of our study treatment (Eckert et al. 2015).

The HAY pens received pelleted timothy hay (Mountain Sunrise Feed, Beryl, UT, USA) in a trough (95.85 L × 15.24 W × 10.16 H cm) placed at the inside back of the pen after pairing. The HAY pens were switched to a long-stem timothy hay from 65 d (± 3 d) until the completion of the trial at 75 d (± 3 d), 7 d postweaning, because of cost constraints, and anecdotally, we have observed that many farmers in the area already start offering timothy hay to their calves at that age. A single box (approximately 22.68 kg) of hay pellets cost $28.35. The cost of feeding the pelleted alfalfa was $1.25/kg as fed. We anticipated each pair would consume 1 box prior to weaning; thus, we implemented a feeding strategy that we believed was economical for farmers to feed their calves ($28.35 for the milk feeding phase). Researchers were not able to be blinded to treatments since the troughs were visible in each pen. All feed was weighed with an OHAUS Valor 4000 Compact Food-Processing Bench Scale (OHAUS Corporation, Parsippany, NJ) before feeding. A maximum of 2 kg of timothy hay pellets were provided to HAY calves daily, and no pairs consumed the entire portion. Feed refusals were weighed daily. All feed was provided at an increasing rate based on consumption to ensure 5% refusals by weight. Calves were given *ad libitum* access to water by bucket from birth. Calf starter that had water sitting on top of the grain and was visibly wet was recorded as missing for the pair for the day. A total of 2% (92/4,480) of data points were excluded from the analysis: 26 points were from the pair that was accidentally fed grain by staff in period 1, 49 from the calves wetting the feed, and 17 because the student forgot to write the grain intake down for the pair that day.

Once a week, a sample of timothy hay pellets, calf starter grain, and a loose hay sample were taken prior to being offered to the calves. Since we sampled from fresh samples offered to the calves, predicted DMI for solid feed was calculated. The samples were placed in a bag with the name of the sample and the date of collection and frozen at −20 °C. Later, each sample was weighed, dried by a forced air oven (VWR Scientific 1690 HAFO Series Oven, Radnor, PA) at 55 °C for 48 h and weighed again to calculate the % DM. After drying, monthly composite samples were made using a Wiley mill (Thomas Scientific, Swedesboro, NJ) and labeled with the contents and date of collection. Samples were sent to Cumberland Valley Analytical Services (Waynesboro, PA, USA) for nutrient analysis, including sugar, NDF, ADF, % CP, % fat, and ash. Calf starter was also evaluated for % starch (Table 1). Feed intake, DMI, and solid FE were calculated at the pen level daily and summarized by period.

**Table 1:** Nutrient composition on a % DM basis for milk replacer, calf starter grain, pelleted timothy hay, and long-stem timothy hay fed to calves.

Nutrient composition, % of DM	Milk replacer	Calf grain (mean ± SEM)	Pelleted timothy hay (mean ± SEM)	Long-stem hay (mean ± SEM)
**ME, Mcal/kg**	4.73	2.13^1^	0.46 ± 0.007	0.46 ± 0.004
**CP**	22.00	23.58 ± 0.37	11.04 ± 0.55	10.62 ± 0.80
**Crude fat**	20.00	3.73^2^	2.57 ± 0.09	3.39 ± 0.31
**ADF**	–	5.58 ± 0.17	38.32 ± 0.67	38.12 ± 1.35
**aNDF**	–	15.66 ± 0.50	59.04 ± 1.73	63.02 ± 1.04
**Starch**	–	32.4 ± 0.50	1.98 ± 0.12	1.86 ± 0.22
**Ash**	–	7.77 ± 0.15	5.28 ± 1.21	7.46 ± 0.47

1 Calculated using standardized values from NASEM (2021).

2 Taken from manufacturer label.

### Body weights

BW were collected from each calf by walking the calf on a halter onto a calibrated scale (Tru-Test EziWeigh 2 scale, Tru-Test Ltd, Hamilton, New Zealand). Calves were weighed at birth, on the days surrounding onset of diarrhea for a concurrent study, then every 3 d for 26 d, followed by every 7 d until study completion on day 75 of age. ADG was calculated between each weight for each calf.

### Health exams

Diarrhea was recorded because it has been associated with reduced weight gain in calves (Schinwald et al. 2022) and was tested as a fixed effect in our models but not retained. Three trained observers performed health exams throughout the trial after obtaining high interobserver reliability (Fleiss kappa interobserver agreement κ  =  0.90). All three researchers were trained by an expert (the PI) for fecal consistency scoring. The three researchers independently scored 40 calves at three separate intervals at one facility to test for interobserver reliability. Fecal consistency was recorded on each calf daily by rectally stimulating the calf to defecate using a thermometer until 30 d of age based on the age of the youngest calf in the pair. The fecal scoring methodology was according to Renaud et al. (2020). A pair had diarrhea if at least one calf had fecal consistency = 3 for 1 d or fecal consistency = 2 for two consecutive days.

Pneumonia has been associated with reduced ADG in calves (Fernandes et al. 2025), so we recorded lung consolidation to test as a potential fixed effect in our models, but it was not retained. Lung ultrasonography was performed on each calf once every 7 d by a single trained observer (achieved Cohen’s kappa = 1.0 with PI before study) after calves were 30 d of age until study completion since this is when calves were observed to be at high risk of pneumonia (Cantor and Costa 2022). Calves were labeled with pneumonia if ≥ 3 cm^2^ of lung consolidation was present according to methods previously described (Cantor and Costa 2022).

### Season

A temperature and humidity logger was placed in the middle of the barn to record ambient temperature, humidity, and THI using the NRC equation (NRC 20001; Kestrel logger, PA, USA). Seasonal thresholds were assigned based on the average THI (according to Ravagnolo and Misztal 2000) in the barn when a calf was in the study. Winter cutoffs were made when the average THI challenged a 12-d old calf’s thermoneutral zone at THI ≤ 50 (as reviewed by Silva and Bittar, 2019) which occurred from February to May 5, 2024 (50.31 ± 6.96; mean ± SD). Summer was when the daily THI exposed calves to heat stress ≥68 (67.56 ± 5.07), and autumn was when the average THI was thermoneutral (58.12 ± 6.37; Silva and Bittar 2019).

### Statistical analysis

All statistical analysis procedures were performed using SAS (version 9.4, Cary, NC, USA). We declared statistical significance at *P* ≤ 0.05. Tendencies were declared at *P *< 0.10. Pairwise comparisons were corrected using Tukey-Kramer’s method. We used *t*-tests (PROC TTEST) to ensure balanced birth weight, serum total protein, and age at pairing among treatments. Data were verified for normality using Shapiro–Wilk’s test and probability distribution plots (PROC UNIVARIATE). Homogeneity of variance was assessed using the Levene test and confirmed. Normality was also confirmed by visually examining the residuals from the linear mixed models and plotting the predicted residuals against the 95% CI. Any suspect outliers outside of the 95% CI from the predicted residuals were tested for model leverage using Cook’s Distance. A Cook’s distance of ≥ 1SD from the other predicted LSM residuals was considered a candidate for removal, but none were observed.

We selected our covariance structures for best model fit using the lowest AIC for Toeplitz, compound symmetry, and first-order autoregressive structures, as well as using goodness-of-fit criteria based on visually inspecting the residuals from the model. Compound symmetry had the best model fit for all outcomes. We forced HAY and period in every model and retained HAY × period interactions at *P *< 0.10. We selected our final multivariable models by stepwise backward elimination. We considered the following variables: calf birthweight (continuous), calf serum total protein (continuous), season (winter, summer, or autumn), calf diarrhea (0.1), and pneumonia (0.1). We also explored antibiotic treatment for any disease (0.1) separately as a fixed effect if diarrhea and pneumonia were not retained. Diarrhea, pneumonia, and antibiotic treatment were not retained in any models. We retained all predictors in our models at a liberal *P *< 0.20.

We used mixed linear regression models (PROC MIXED) to assess the effect of HAY and the HAY × period interaction with calf BW, and calf ADG. Hay, the HAY × period interaction, period, season, and birthweight were retained. Serum total protein was also retained in the calf BW model. We used period as a repeated measure with calf as the subject and used pair nested within block as a random effect.

Researchers were responsible for feeding calves daily. However, because of a communication error, one CON pair was accidentally fed calf starter by the farm staff for one week in period 1. Because we were not confident in this CON pair’s calf starter intake, we omitted it for period 1. Because feed intakes were recorded and assigned at the pen level, individual covariates by calf were averaged by pair and were assigned at the pen level. Mixed linear regression models (PROC MIXED) were used to assess the effect of HAY and the HAY × period interaction with paired calf starter DMI and solid FE. Hay, the HAY × period interaction, pair BW, and period were retained. We used period as a repeated measure with pair as the subject and nested pair within block as a random effect. Solid FE was calculated as the ratio of ADG to total DMI (= paired calf starter DMI + paired hay DMI) by period according to the equation below:


$$\mathrm{Solid}\;\mathrm{FE}=(\frac{\mathrm{ADG}}{\mathrm{DMI}})$$

## Results

### Descriptive statistics

The average age at pairing did not differ between treatments (CON 5 ± 2 d vs. HAY 5 ± 3 d; *P *= 0.81; mean ± SD). The minimum age at pairing for a calf was 2 d of age and the maximum was 14 d of age. Pair birthweights were not different (CON 39.31 ± 2.31 kg vs. HAY 40.78 ± 2.18 kg; *P *= 0.13) and ranged from 33.1 to 49.0 kg. Pair serum total protein was also not different (CON 5.88 ± 0.33 g/dL vs. HAY 5.97 ± 0.45 g/dL; *P *= 0.62) and ranged from 5.1 to 7.7 g/dL. 81.3% (13/16) of CON and 87.5% (14/16) of HAY pairs had at least one calf in a pair have diarrhea throughout the study. 43.8% (7/16) of CON and 50% (8/16) of HAY pairs had at least one calf have ≥ 3cm^2^ of lung consolidation in the pair throughout the study. All calves completed the study.

### BW and ADG

**Figure 1:**
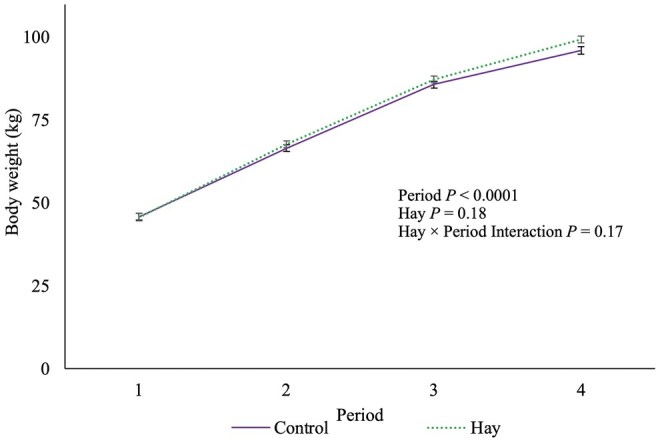
The association of period, pelleted timothy hay or a negative control, and the hay × period interaction with body weight (LSM ± SEM) of 64 paired Holstein heifer calves using a mixed linear regression model (16 pairs per treatment). We used periods to divide weight gain into biologically relevant growth periods. Period 1 = birth to day 26, period 2 = day 27 to 60, period 3 = day 61 to 67, and period 4 = day 68 to day 75 ± 3 d of age.

There was no HAY × period interaction observed for calf BW (*P *= 0.17; Figure 1). Hay was also not associated with calf BW (*P *= 0.18). Serum total protein tended to be positively associated with calf BW (*P *= 0.07). Period and calf birthweight were positively associated with bodyweight gain (*P *≤ 0.0001). There was a tendency for season to be associated with calf BW (*P *= 0.10), with winter calves tending to weigh −3.48 ± 1.67 kg less than calves born in autumn (*P *= 0.10); no other differences among seasons were observed (*P *≥ 0.21). On average, calves in the winter weighed 72.39 ± 1.18 kg, in the summer they weighed 74.66 ± 0.70 and in autumn they weighed 75.87 ± 1.18 kg.

There was no HAY × period interaction observed for ADG (*P *= 0.75; Table 2). There was no association of HAY (*P *= 0.97) or season (*P *= 0.17) with calf ADG, but period was positively associated with ADG (*P* < 0.0001), and birthweight tended to be positively associated with calf ADG (*P *= 0.06). The ADG was (CON 0.88 ± 0.03 kg/d vs. HAY 0.88 ± 0.03 kg/d).

**Table 2:** The effect of feeding a pelleted timothy hay, or a negative control, period, and the hay × period interaction on the average daily gains and feed efficiency (LSM ± SEM) of 32 pairs of Holstein heifer calves using a mixed linear regression model. The interactions were adjusted with Tukey corrections.

	Treatment				*P–*values fixed effects		
Variable	CON (LSM)	SEM	HAY (LSM)	SEM	Treatment	Period**^1^**	Treatment × period
**Overall ADG, kg/d**	0.88	0.03	0.88	0.03	0.97	<0.0001	0.75
**Overall solid FE**	0.65	0.07	0.73	0.07	0.38	<0.0001	0.90
**Period 1**							
** ADG, kg/d**	0.52	0.05	0.47	0.05			1.00
** Solid FE**	1.61	0.10	1.68	0.10			1.00
**Period 2**							
** ADG, kg/d**	0.83	0.05	0.85	0.05			1.00
** Solid FE**	0.57	0.10	0.61	0.10			1.00
**Period 3**							
** ADG, kg/d**	0.95	0.05	0.92	0.05			1.00
** Solid FE**	0.24	0.10	0.33	0.10			1.00
**Period 4**							
** ADG, kg/d**	1.21	0.05	1.27	0.05			1.00
** Solid FE**	0.16	0.10	0.31	0.10			0.95

1 The periods were period 1 (0 to 21 d), period 2 = (22 to 55 d), period 3 weaning = (56 to 62 d), and period 4 after weaning = (63 to 70 d). Days on the study refer to days after pairing.

### Calf starter DMI, hay DMI, and solid FE

**Figure 2:**
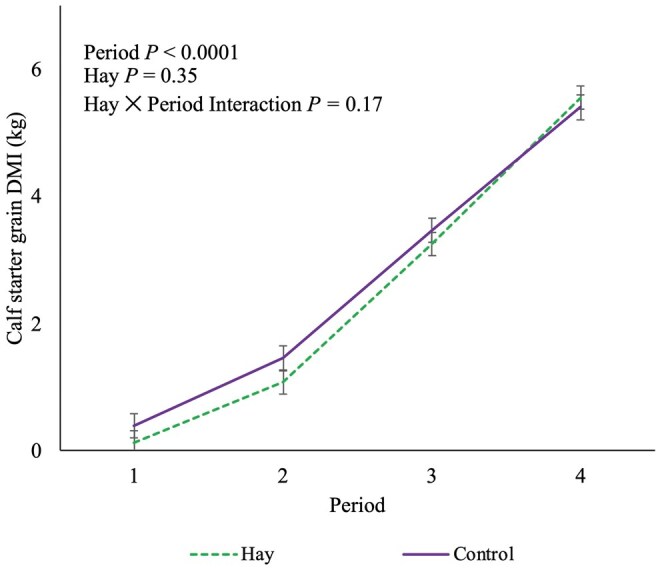
The association of period, pelleted timothy hay or a negative control and the hay × period interaction with daily calf starter dry matter intake (LSM ± SEM) for 64 paired Holstein heifer calves using a mixed linear regression model (16 pairs per treatment). We used periods to divide weight gain into biologically relevant growth periods. Period 1 = birth to day 26, period 2 = day 27 to 60, period 3 = day 61 to 67, and period 4 = day 68 to day 75 ± 3 d of age.

**Figure 3:**
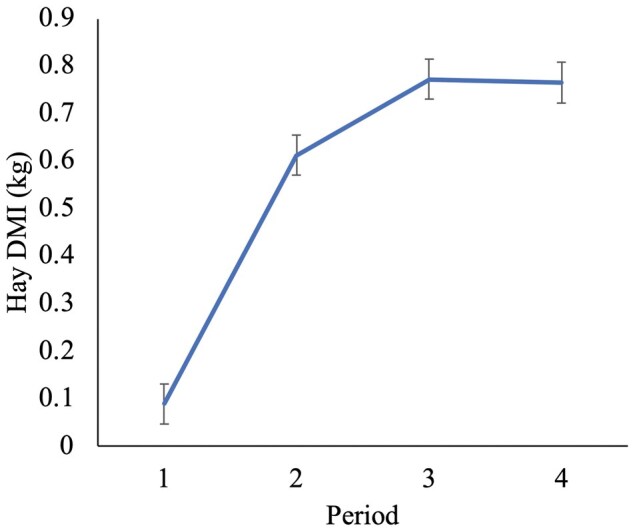
The intake of timothy hay (*n* = 16 pairs, mean ± SD) of Holstein heifer calves (e.g., pelleted hay on calf performance outcomes). Period 1 = birth to day 26, period 2 = day 27 to 60, period 3 = day 61 to 67, and period 4 = day 68 to 75 ± 3 d of age.

Because the calf bottle starter intakes were negligible, we merged the starter intake data from the bucket with the bottle. There was no association of HAY (*P *= 0.35) with calf starter DMI. There was an association of period (*P *< 0.0001) and pair birthweight (*P *= 0.001) with overall DMI of calf starter (CON 2.68 ± 0.15 kg vs. HAY 2.50 ± 0.14 kg/d). There was no HAY × period interaction observed for calf starter DMI (*P *= 0.17; Figure 2). The DMI for HAY calves is shown in Figure 3. There was no association of HAY (*P *= 0.41) or pair serum total protein (*P *= 0.20), only period (*P *< 0.0001) with solid FE (CON 0.65 ± 0.07 vs. HAY 0.73 ± 0.07; *P *= 0.41). There was no HAY × period interaction for solid FE (Table 2; *P *= 0.62).

## Discussion

Previous work has found no major benefits of offering pelleted alfalfa to calves (Jahani-Moghadam et al. 2015; Molaei et al. 2021), and we also observed no effect of feeding pelleted timothy on calf performance, DMI, or FE. The results from our study that offered calves pelleted timothy hay agree with previous studies feeding pelleted alfalfa hay as part of the total mixed ration, which did not improve calf growth performance (Jahani-Moghadam et al. 2015; Molaei et al. 2021). Our results disagreed with findings from studies that offered calves long-stem hay who observed increased growth performance in young calves (Khan et al. 2011; as reviewed by Khan et al. 2016). We suspect that our results differed from Khan’s work because they fed long-stem hay. Long-stem hay reduces abnormal oral behaviors in calves (Horvath and Miller-Cushon, 2019; Zhang et al. 2021). This discourages cross-sucking by prolonging eating and chewing activities and enhancing ruminal fill and buffering. Pelleted hay contains more nutrients per gram than long-stem hay, likely being digested much quicker than long-stem hay, decreasing ruminal retention and buffering effects (NASEM 2021). Moreover, we offered calves a teat enrichment with grain, which may have redirected oral behavior away from the pelleted hay. Others have found that offering grain enrichments with a teat decreased abnormal oral behavior in paired calves (Salter et al. 2021). We hypothesize that it is possible that providing pelleted timothy hay to paired calves in addition to offering a teat as an outlet for non-nutritive oral behavior may have been why we did not observe any association of hay with calf performance. In a companion study, we observed that there was no association of pelleted hay with the latency to start cross-sucking or the duration of bouts surrounding the 6 h/d that the calves drank milk (Plaugher and Cantor 2025). We suggest that in the absence of suckling outlets such as a Braden bottle, social facilitation likely promotes hay intake as a primary medium for oral manipulation in paired calves; this potential relationship warrants further investigation.

Our pair-housed hay calves had similar growth performance to previous studies that used individually housed calves offered alfalfa pellets (Jahani-Moghadam et al. 2015; Molaei et al. 2021) and those that were offered 4.0 L/d of MR (Kincaid 1980). Jahani-Moghadam et al. (2015) found no difference in growth performance for calves fed higher planes of milk nutrition and offered pelleted or chopped alfalfa versus controls. This is probably due to the variety of forage fed, as a meta-analysis that included long-stem and pelleted alfalfa research found an association of alfalfa feeding with decreased growth performance when compared to other varieties of hay (Imani et al. 2017). Kincaid (1980) found that calves offered pelleted alfalfa at varying inclusion rates and free-choice long-stem alfalfa had significantly greater total feed intakes than controls not offered hay. However, pelleted hay calves in that study had decreased starter intakes vs. calves offered long-stem alfalfa, or controls, suggesting that even alfalfa pelleted hay does not offer superior performance benefits over long-stem hay (Kincaid 1980). The decreased level of concentrate intake in calves fed *ad libitum* pellets in Kincaid (1980)’s study is likely due to the limited amount of milk being fed (e.g., <0.5 kg/d) as the calves were likely hungry and more motivated to eat pelleted hay than concentrate. Molaei et al. (2021) did not have any control calves in their study, so it is difficult to compare their findings to ours. However, offering a pelleted timothy hay in this study did not decrease solid feed DMI, which has been observed by others who offered calves long-stem bromegrass and orchardgrass (Coverdale et al. 2004; Castells et al. 2012). We suggest research is still lacking on the effects of long-stem timothy hay feeding on paired calf performance, and this work is warranted.

There was no effect of HAY on ADG in our study. The effect of period on ADG was expected as the calves grew over time. Starter DMI increased linearly over time, and we found no effects of HAY on starter grain intake, which agrees with Jahani-Moghadam et al. (2015), who also fed pelleted hay. This is an important finding, as the provision of long-stem and chopped hay has previously been discouraged due to the potential for gut fill to limit concentrate intake (Drackley 2008; Hill et al. 2008). The intake of starter grain during the preweaning period is important for rumen development and for creating a smooth transition to solid feed during weaning (Roth et al. 2009). We suspect that we did not observe differences in ADG because we offered this to calves as a pellet. Others have observed performance benefits to offering long-stem and chopped varieties of grass hay to calves (Khan et al. 2011; Khan et al. 2016). However, it was outside the scope of our study to investigate if pelleted hay improved rumen performance. Since ADG was not positively impacted by hay, more research is needed to justify feeding pelleted hay.

We did not observe an association of HAY with calf solid FE, which is different than others, likely because we offered calves timothy hay at a maximum inclusion rate in the diet of 2.0 kg/d. For example, Hill et al. (2008) found that the inclusion of long-stem hay fed *ad libitum* in preweaned calf diets decreased FE in individually housed Holstein bull calves offered <0.5 kg/d of MR. Castells et al. (2012) found no association of offering grass hay with FE, which they hypothesized was due to similar DM digestibility between treatments in individually housed calves. Our results agree with Castells et al. (2012), likely because we also offered a grass hay variety to our calves. Coverdale et al. (2004) found improved FE after weaning in calves offered a bromegrass hay preweaning, suggesting that the provision of forage during the preweaning period may have prepared the rumen to better utilize feeds during the postweaning period. Further research should investigate biological differences that may affect FE in pair-housed calves and the effects of different hay varieties on DM digestibility, as we hypothesize that these may be important factors in paired calf FE. Notably, we observed no association of HAY with calf starter DMI and pairs were all step-down weaned across 7 d.

There were limitations in this study. Forage sampling of the loose hay that was fed around weaning in this study was not done with core bale sampling. Core bale sampling is the ideal technique to capture the true nutrient composition of long-stem hay. To attempt to circumvent this limitation, long-stem hay was sampled weekly. Additionally, the nutrient composition of the long-stem hay was closely matched to the pelleted hay to ensure consistency. This resulted in very little differentiation in nutrient content between the long-stem and pelleted timothy hay. Another consideration was the length of time (e.g., 7 d) that calves were monitored postweaning. Calves were not followed for a longer time because of labor and barn space limitations. However, it is possible that calves offered the forage may have experienced growth benefits had they been followed longer postweaning. For example, a meta-analysis investigating the effects of forage feeding on calf performance found that providing at least 10% DM of the diet as a forage source to calves improved BW gains; in fact, 70% (17/24) of these studies followed calves for at least 10 d post-weaning (Imani et al. 2017). The BW gain benefits were dependent on if ground or texturized calf starter was fed, and it was suggested that ruminal fill accounted for the BW differences (Imani et al. 2017). Indeed, it is possible that the HAY calves had a better prepared rumen than the control calves, and perhaps growth benefits would become evident further into the postweaning period. We suggest that future work should follow paired dairy calves to investigate the association of loose timothy hay feeding with performance outcomes for at least 14 d after weaning.

## Conclusion

Offering pelleted timothy hay to preweaned pair-housed calves offered 1.0 kg/d of MR powder did not positively impact ADG, BW, solid feed DMI, or FE in paired dairy calves up to 75 (± 3) d of age compared to negative controls. We suggest that pelleted timothy hay provides an additional source of fiber to calves without compromising performance, but more research is needed regarding rumen development to justify producers switching to pelleted hay.

## Abbreviations

ADF: acid detergent fiber
ADG: average daily gain
BW: body weight
CP: crude protein
DM: dry matter
DMI: dry matter intake
FE: feed efficiency
MR: milk replacer
NDF: neutral detergent fiber
THI: temperature humidity index
